# Morphologic Pattern Differences in Reconstructive Tissue Repair of Bone Defects Mediated by Bioactive Ceramics and Hydrogels: A Microscopic Follow-Up Evaluation of Re-Ossification

**DOI:** 10.3390/gels11070529

**Published:** 2025-07-09

**Authors:** Róbert Boda, Viktória Hegedűs, Sándor Manó, Andrea Keczánné-Üveges, Balázs Dezső, Csaba Hegedűs

**Affiliations:** 1 Department of Oral and Maxillofacial Surgery, Faculty of Dentistry, University of Debrecen, 4032 Debrecen, Hungary; boda.robert@dental.unideb.hu; 2 Department of Pediatric Dentistry and Orthodontics, Faculty of Dentistry, University of Debrecen, 4032 Debrecen, Hungary; 3 Department of Orthopedics, University of Debrecen, 4032 Debrecen, Hungary; 4 Department of Biomaterials and Prosthetic Dentistry, Faculty of Dentistry, University of Debrecen, 4032 Debrecen, Hungary; 5 Department of Oral Pathology and Microbiology, Faculty of Dentistry, University of Debrecen, 4032 Debrecen, Hungary; 6 Department of Pathology, Faculty of Medicine, University of Debrecen, 4032 Debrecen, Hungary

**Keywords:** bio-ceramics, βTCP-aerogel, βTCP-hydrogel, granulomatous inflammation, osteo-induction, tissue remodeling

## Abstract

Although publications have documented the osteo-inductive effects of various bioactive materials on tissue sections, the associated morphologic patterns of tissue remodeling pathways at the cellular level have not been detailed. Therefore, we present a comparative histopathological follow-up evaluation of bone defect repair mediated by silica aerogels and methacrylate hydrogels over a 6-month period, which is the widely accepted time course for complete resolution. Time-dependent microscopic analysis was conducted using the “critical size model”. In untreated rat calvaria bone defects (control), re-ossification exclusively started at the lateral regions from the edges of the remaining bone. At the 6th month, only a few new bones were formed, which were independent of the lateral ossification. The overall ossification resulted in a 57% osseous encroachment of the defect. In contrast, aerogels (AE), hydrogels (H), and their β-tricalcium-phosphate (βTCP)-containing counterparts, which were used to fill the bone defects, characteristically induced rapid early ossification starting from the 1st month. This was accompanied by fibrous granulomatous inflammation with multinucleated giant macrophages, which persisted in decreasing intensity throughout the observational time. In addition to lateral ossification, multiple and intense intralesional osseous foci developed as early as the 1st month, and grew progressively thereafter, reflecting the osteo-inductive effects of all compounds. However, both βTCP-containing bone substituents generated larger amounts and more mature new bones inside the defects. Nevertheless, only 72.8–76.9% of the bone defects treated with AE and H and 80.5–82.9% of those treated with βTCP-containing counterparts were re-ossified by the 6th month. Remarkably, by this time, some intra-osseous hydrogels were found, and traces of silica from AE were still detectable, indicating these as the causative agents for the persistent osseous–fibrous granulomatous inflammation. When silica or methacrylate-based bone substituents are used, chronic ossifying fibrous granulomatous inflammation develops. Although 100% re-ossification takes more than 6 months, by this time, the degree of osteo-fibrous solidification provides functionally well-suited bone repair.

## 1. Introduction

To facilitate bone regeneration, reconstructive surgery and dental implantology often use high-performance tissue-friendly, non-toxic, reliable, and reproducible artificial solid or granular- or nano-particle-containing exogenous materials. When grafted appropriately, these materials are able to integrate stably and avidly into the host’s bone defect (e.g., titanium implants) or provide a temporal scaffolding with solidification while stimulating the physiological processes of re-ossification for complete repair [1,2,3].

**Figure 1:**
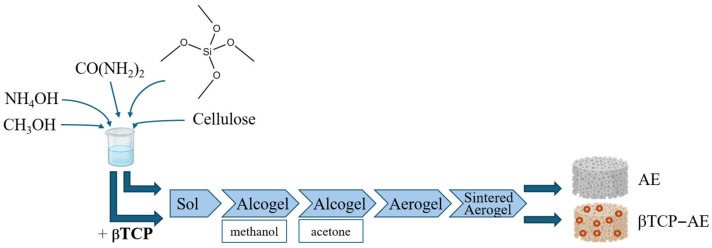
Schematic illustration of the preparation of aerogels for rat calvaria bone defect studies.

Recent advances in bone tissue engineering have increasingly focused on aerogels as highly promising bioactive ceramic scaffolds due to their ultralight, highly porous, and biomimetic extracellular matrix-like structures. This group includes biocompatible silica-based materials that may contain calcium phosphates, the most prominent bone matrix components at the molecular level, with osteo-inductive features [4,5,6]. These composites, synthesized via sol-gel techniques and supercritical drying, exhibit mesoporous structures with interconnected pores that facilitate nutrient transport and cell infiltration. A general schematic illustration of the multi-step preparation of an aerogel and a β-tricalcium phosphate-containing silica-based aerogel is shown in Figure 1.

Importantly, heat treatment of these aerogels at moderate temperatures (700–800 °C) optimizes their mechanical properties, porosity, and dissolution behavior, releasing calcium and phosphate ions in ratios favorable for bone tissue induction [7]. Similarly, hydroxyapatite nanowire-based aerogels have been shown to possess ultrahigh porosity (~98.5%) and excellent elasticity, promoting mesenchymal stem cell adhesion, proliferation, and differentiation, which accelerates bone regeneration and neovascularization in vivo [8]. Composite aerogels incorporating bioactive ions, such as Zn^2+^ and Si^4+^, combined with anti-inflammatory agents, such as aspirin, have demonstrated enhanced biocompatibility, vascularization, and osteoblast differentiation, effectively promoting in situ bone defect repair while modulating local inflammatory responses [9]. Furthermore, hybrid nanofiber aerogels loaded with osteoinductive peptides, such as BMP-2, have achieved significant improvements in cranial bone healing and vascularization, highlighting the potential of aerogels as multifunctional scaffolds capable of sustained therapeutic delivery [10].

**Figure 2:**
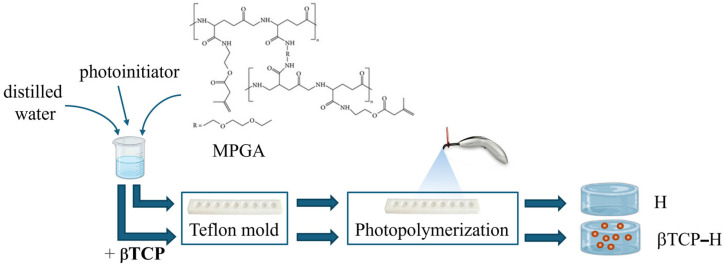
Schematic illustration of the preparation of hydrogels for rat calvaria bone defect studies.

In parallel, hydrogels have emerged as versatile biomaterials that mimic the hydrated extracellular matrix, offering a conducive environment for cell proliferation, differentiation, and nutrient exchange essential for bone regeneration. Injectable hydrogels enable minimally invasive delivery of bioactive molecules, osteogenic growth factors, and stem cells, making them especially suitable for irregular or non-load-bearing bone defects [11]. Recent developments in nanocomposite hydrogels, such as those incorporating guanidinylated hyaluronic acid and silica-rich nanoclays, have demonstrated self-healing properties and enhanced osteogenic capacity by effectively delivering demineralized bone matrix particles in vivo [12]. The tunable mechanical properties and dynamic crosslinking of these hydrogels facilitate sustained bioactivity and integration with host tissue, addressing challenges related to particle dispersion and osteo-inductivity. The simplified preparation scheme of a methacrylated poly-γ-glutamic acid (MPGA)-based hydrogel (HG) [13] and its composite version containing βTCP (βTCP-HG) is shown in Figure 2.

Together, aerogels and hydrogels represent complementary strategies in bone tissue engineering: aerogels provide structural support and osteoconductivity through their porous ceramic frameworks, while hydrogels offer biological mimicry and controlled delivery capabilities, advancing the development of effective bone regeneration therapies [5,14].

Material scientists continue to innovate and develop new biocompatible synthetic bone grafting materials to improve the quality of treatment in orthopedic and plastic surgeries, as well as dental implant management, with widespread ongoing corresponding basic research aimed at optimizing physicochemical properties. However, in vivo follow-up data remain limited on the in situ role of such grafts at the cellular tissue sites of application, specifically regarding the interface region and defect interior, to assess patterns of remodeling using conventional microscopic analyses. 

This report presents a detailed, concise comparative microscopic follow-up evaluation with chronological photo documentation, demonstrating the various morphological and cellular tissue patterns observed during bone defect repair. These observations are correlated with the presence of mesoporous silica aerogel (AE), β-tricalcium phosphate (βTCP)-containing AE (βTCP-AE), hydrogel (H), and βTCP-hydrogel (βTCP-H) in an experimental rat calvaria bone defect model.

## 2. Results and Discussion

### 2.1. Results

The main aim of the present study was to carry out a comparative microscopic follow-up evaluation of in vivo bone defect reconstruction at the cellular level, focusing on the time-dependent healing course within the lesion when using these bioactive materials for bone grafting.

#### 2.1.1. Comparative Chronological Histopathology of AE and βTCP-AE Scaffolds at 1, 3, and 6 Months Following Implant Treatments of Calvaria Bone Defects in Rats

**Figure 3:**
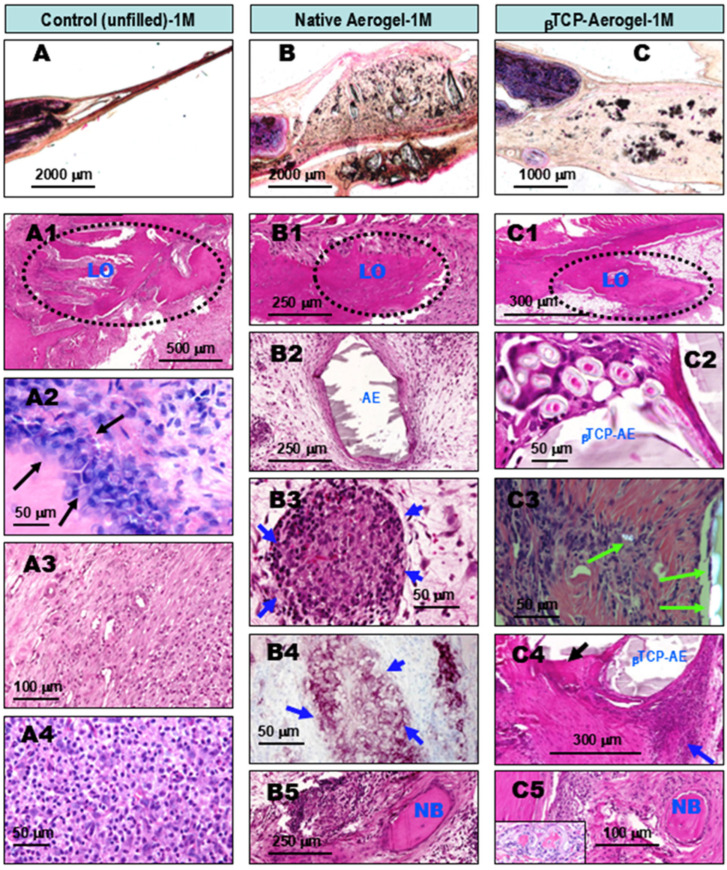
Comparative histopathology of wound healing in rat calvaria bone defects, one month (1M) after operation. The defects were either left untreated (control, left column), grafted with silica aerogel (AE, middle column), or filled with βTCP-aerogel (βTCP-AE, right column). The upper panel images (**A**–**C**) show hematoxylin–eosin (HE)-stained ground sections of resin-embedded native, non-decalcified specimens, providing a view of one of the bone defect’s edges (the black discolorations are deposited artifacts from the polishing procedure). All other images show HE-stained and decalcified tissue samples. As shown in the representative images of the untreated control specimen (**A1**–**A4**), the lateral edge of the bone defect exhibited reparative ossification with a budding pattern (**A1**, LO), accompanied by osteoblast proliferation (**A2**, arrows). The rest of the bone defect exhibited fibrous granulation tissue with the presence of leukocytes (**A3**,**A4**). In contrast to the control-comparable LO (**B1**,**C1**), the presence of silica components in the aerogels led to the development of chronic fibrous granulomatous inflammation in both the AE- and the βTCP-AE (**B2**,**C2**) treated bone defects. As a result, granulomas formed with the presence of epitheloid (activated) cell clusters (**B3**,**C4**, blue arrows), which were characteristically immune-positive for CPM (**B4**, purple cells, blue arrows). Crystalline silica particles were identified in both groups, showing typical birefringence under a polarizing microscope, as marked in (**C3**) with green arrows. Remarkably and most importantly, both the AE- and βTCP-AE treated bone defects exhibited early intralesional ossifications with new bone formation and osteoid formations inside the defect, independent of lateral ossification (**B5**,**C5**, NB). This was preceded by calcification (**C4**, black arrow). LO, lateral ossification (arising from the edge of the bone defect); AE, silica aerogel; βTCP-AE, β-Tricalcium phosphate silica aerogel; NB, new bone.

**At the 1st month of observation after implant treatment with silica-based AE and βTCP-AE materials,** early lateral re-ossification was observed in all specimens. AE-mediated foreign body granulomatous inflammation was also observed, with intra-lesional calcification or early ossification (Figure 3). The reference control tissues where bone defects were left unfilled with any alloplastic materials showed reparative ossifying processes arising exclusively from the lateral edges of the remaining bone by the first month (Figure 3A,A1, within the dotted area). This was accompanied by osteoblast proliferation (Figure 3A2, arrows) surrounded by fibrous granulation tissue (Figure 3A3) that was rich in leukocytes (Figure 3A4). However, no intralesional bone formation was noted within the central regions in the control animals by this time, indicating that this occurred independently from lateral ossification. In contrast, in addition to the control-comparable lateral bone formations in calvaria bone defects grafted with aerogel (AE) or βTCP-AE (Figure 3B,B1,C,C1, within the dotted circles), both exogenous materials were found to precipitate in large foci demarcated by fibrous granulation tissue (GT) (Figure 3B2,C2, with AE and βTCP-AE indications). These areas were accompanied by chronic active granulomatous inflammation (Figure 3B3) with the presence of epithelioid macrophage clusters (Figure 3B3,C4, blue arrows). These cluster cells were identified using a characteristic marker, anti-CPM (carboxypeptidase M) antibody, by means of immunocytochemistry (Figure 3B4, purple cells). Additionally, some of the silica aerogel substances of both the AE and the βTCP-AE were also clearly identified under a polarizing microscope, as shown in Figure 3C3 image (green double arrows), with some particles ingested by macrophages (single green arrow), reflecting the crystal form of the silica. It is noteworthy that both the AE- and βTCP-AE-treated bone defects showed early intralesional calcifications (Figure 3C4, black arrow) and islets of new bone formations (NB) within the internal regions of the defect (Figure 3B5,C5), indicating osteo-inductions regardless of the ongoing lateral osseous regeneration along the defects’ bony borders.

**Figure 4:**
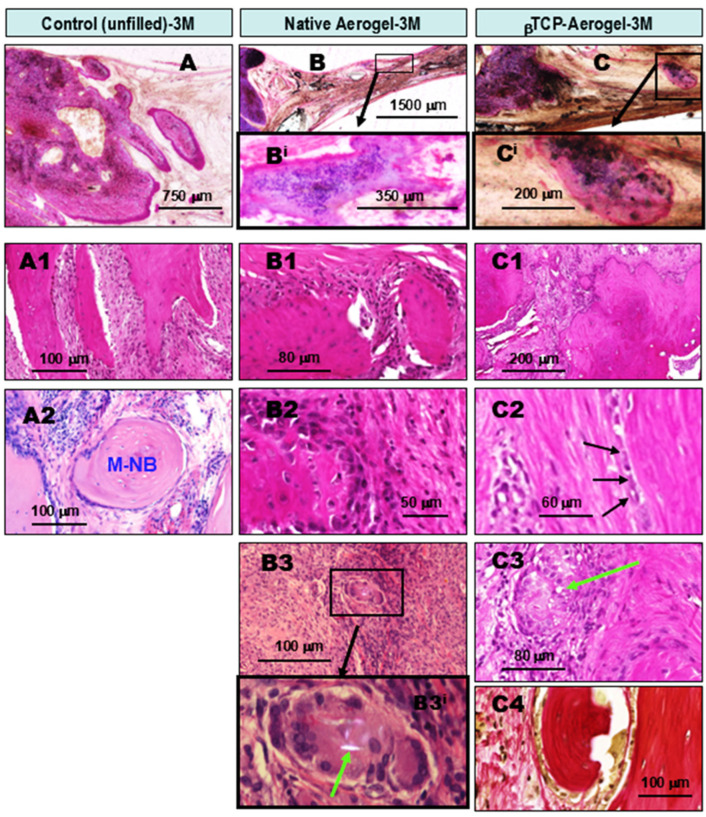
Comparative histopathology of wound healing in rat calvaria bone defects, three months (3M) after operation. The defects were either left untreated (control, **left column**) or grafted with silica AE (**middle column**) or βTCP-AE (**right column**). The upper images (**A**–**C**), and (**B**,**C**)’s magnified regions, (**B^i^**,**C^i^**) depict HE-stained sections of non-decalcified ground specimens for a view of one of the bone defect’s edges and inside the lesion. The untreated control bone defect showed further progression in lateral ossification, accompanied by fibrosis and mononuclear inflammatory cells (**A**,**A1**), and some of the newly formed bone became nearly mature (**A2**, M-NB). Basically, no intralesional bone formation could be found at this time, but calcifications were identified within the solidified scar tissue inside the bone defect. In contrast, all of the AE- and βTCP-AE-treated bone defects exhibited both lateral ossification (**B**,**B1**,**C**,**C1**) and intralesional calcification (**B^i^**). In addition, early new bone formations were observed, accompanied by active osteoblast proliferation (**B2** left, **C^i^**, **C2**, arrows) within the fibrous granulomatous inflammatory tissue. Occasionally, multinucleated giant macrophages were present, which engulfed AE silica crystal particles (**B3**, **C3**, green arrow). The digitally magnified image in (**B3^i^**) presents a multinucleated giant macrophage that ingested a silica crystal particle (green arrow). Although most of the newly formed bones had immature osteoid matrices at this time (3M), few readily exhibited a lamellar structure, indicating a mature organoid compact bone pattern (**C4**). (**C4**) presents a van Gieson (vG)-stained section. All other images present HE-stained sections.

**At the 3rd month of observation after implant treatment with AE and βTCP-AE silica materials,** ongoing lateral ossification was observed in all groups. Progressive multidirectional new bone formations were also observed inside the lesions in the AE- and βTCP-AE-treated tissue samples (Figure 4). The untreated control bone defect showed further progression of lateral ossification with a budding pattern (Figure 4A) and a fibrous granulation tissue border (Figure 4A1), while some newly formed bone was partially maturing (Figure 4A2, M-NB). However, intralesional bone formation in the control specimens, independent from the lateral regenerative ossification, was still not apparent, although some focal calcifications were observed. In contrast, all of the AE- and βTCP-AE-treated bone defects exhibited both lateral ossification (Figure 4B,B1,C,C1) and intralesional calcifications (Figure 4B^i^). Additionally, new bone formations accompanied by active osteoblast proliferation (Figure 4B2,C^i^,C2, arrows) appeared, along with fibrous granulomatous inflammation and the presence of multinucleated giant macrophages that engulfed AE particles (Figure 4B3,C3, arrow). The digitally magnified large cell using polarizing microscopy, shown in Figure 4B3^i^, demonstrates a multinucleated giant macrophage that ingested a silica crystal particle (green arrow). Although most of the newly formed bones exhibited immature osteoid matrices at this time (3M), which turned out to not be amyloid, a few showed a lamellar structure, indicating an organoid maturing pattern (Figure 4C4).

**Figure 5:**
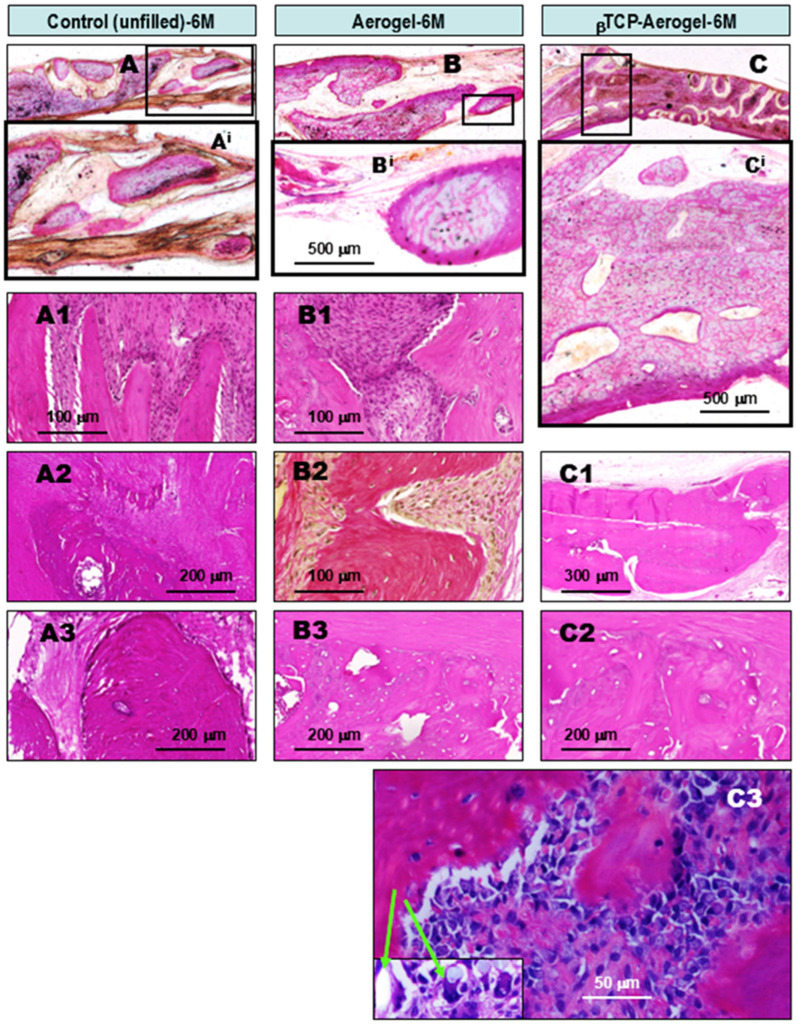
Comparative histopathology of rat calvaria bone defect repair, 6 months (6M) after operation. The defects were left untreated (control, **left column**) or grafted with silica AE (**middle column**) or βTCP-AE (**right column**). The upper pictures (**A**–**C**) depict HE-stained ground (frontal) sections for an overview of the lesion, and their framed regions show detailed, magnified images (**A^i^**,**B^i^**,**C^i^**). The untreated bone defects exhibited advanced lateral ossifications within inflammatory, dense, fibrous, and scarred solidified tissues (**A1**, left, and bottom pink spikes protruding upward), along with intralesional calcifications (**A2**) and ossification (**A3**), which were morphologically reminiscent of dystrophic calcification–ossification (**A2**,**A3**). In addition to the control-comparable lateral ossification in both the silica AE- and βTCP-AE-treated bone defects, large amounts of coalescent, newly formed compact bone tissue was observed inside the bone defects (**B3**,**C1**,**C2**). As shown in (**B1**,**B2**) (van Gieson-stained) sections, between the newly formed and maturing bones (**B1**, left and right pink area; **B2**, central yellowish-red lamellated hourglass-shaped parts), small foci of ongoing low-grade chronic fibrous inflammation were present, with accumulated collagen bundles (**B2**, at left and right red fibers). These areas were partly associated with the presence of remaining non-metabolized silica crystals from the aerogel, detected under a polarizing microscope (**C3**, insert, green arrows). Apart from (**B2**), all images depict HE-stained sections.

**At the 6th month of observation after implant treatment with AE and βTCP-AE silica materials,** advanced narrowing of the defect was observed, with lateral osseous reconstruction present in all groups. Additionally, substantial, well-developed large central bony solidification was observed in βTCP-AE-treated specimens, and to a lesser extent in AE-treated specimens (Figure 5). In the control samples, large, thick, and partially coalescent lateral ossification was observed, accompanied by chronic active fibrous inflammation (Figure 5A,A^i^,A1), which narrowed the defect considerably but not completely. At this stage, the remaining central area of the bone defect exhibited fibrous tissue and showed calcifications with osseous metaplasia comparable with dystrophic ossification (Figure 5A2,A3). In contrast, alongside lateral ossification comparable or greater than that in the control, both the silica aerogel (AE)- and βTCP-AE-treated bone defect specimens (Figure 5B,B^i^,C,C^i^) demonstrated advanced solid organoid bony coalescent tissues in large amounts within the central areas of the lesion, as well decreased osteoblastic activities by the 6th month of the observational period (Figure 5B3,C1,C2). These were partially embedded in dense fibrous soft tissue (Figure 5B1,B2), with low-grade chronic inflammation. Nevertheless, complete (100%) ossification was not observed in these cases either, although the overall amount of new bone observed was much higher, especially in the βTCP-AE-treated bone defect cases, as compared to the untreated control specimens. However, it is noteworthy that tiny remnants of unmetabolized silica crystals could be detected by means of polarizing microscopy both in the βTCP-AE grafted specimens (Figure 5C3, insert, arrows) and the AE samples, reflecting persistent granulomatous inflammation even at the 6th month, the final observation time.

#### 2.1.2. Comparative Chronological Histopathology of H and βTCP-H Scaffolds at 1, 3, and 6 Months Following Implant Treatments of Calvaria Bone Defects in Rats

**Figure 6:**
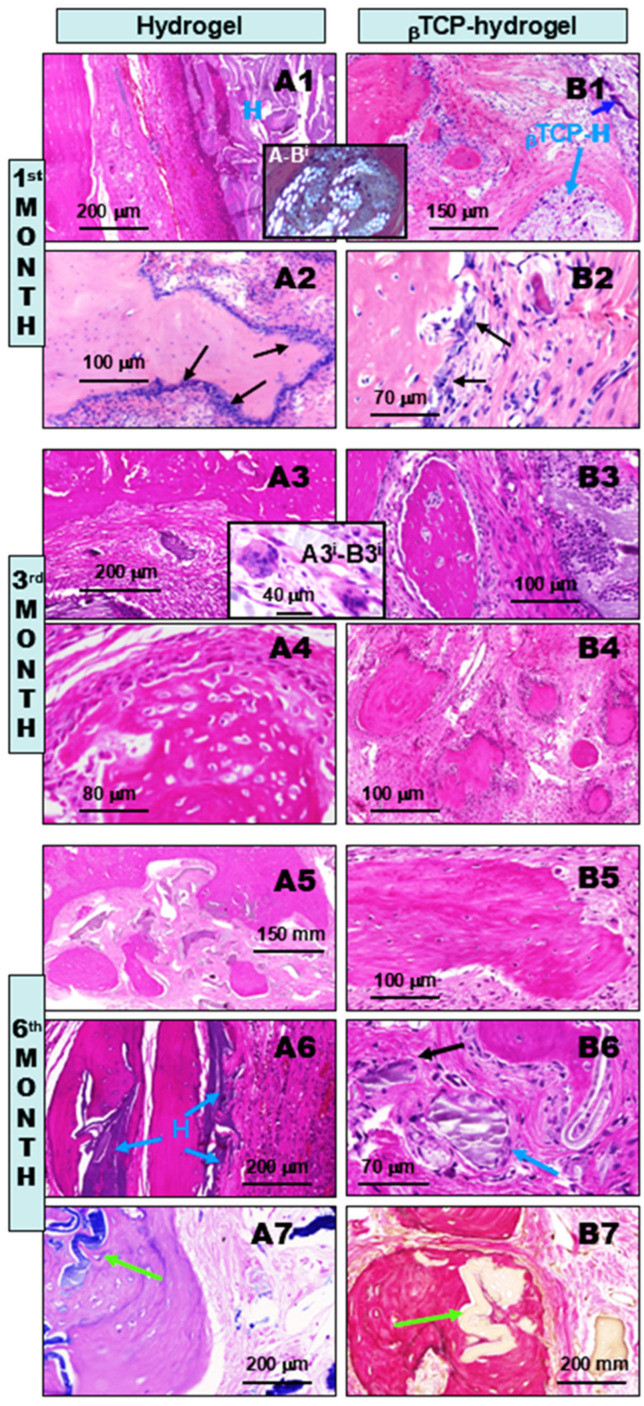
Histological follow-up of rat calvaria bone defect repair after hydrogel (**left column**, **A1**–**A7**) and βTCP-hydrogel treatments (**B1**–**B7**) over a six-month postoperative period. During the 1st month, large pools of hydrogel (H) (**A1**, right) and β-tricalcium phosphate-hydrogel (βTCP-H) (**B1**, right, arrow) were observed, exhibiting characteristic birefringence under polarizing microscopy due to the methacrylate contents (insert **A**-**B^i^**, bright materials). This induced chronic granulomatous inflammation, accompanied by osteoblast proliferation (**A2**,**B2**, arrows), leading to new bone formation both at the lateral regions (**A1** left; **B1** left upper) and inside the defect (**B1** middle), where foci of calcifications were also found (**B1** dark blue arrow; **B2** right upper). At the 3rd month, in addition to the presence of non-metabolized H (**A3** left lower corner; **B3** right), both groups exhibited progressive multifocal. These appeared to be most prominent in the βTCP-H group (**B3**,**B4**), though both groups exhibited multinucleated giant cells (**A3**,**B3**, insert), accompanied by methacrylate-based H-induced persistent granulomas. At the 6th month postoperatively, ongoing ossifications were observed in both groups (**A5**–**A7**,**B5**–**B7**), with the maturation of new bone islets characterized by lamellar osseous tissue formation (**B5**,**A7**,**B7**). Remarkably, some remnants of the H and βTCP-H were found inside the newly formed bone tissue, identifiable as blue in Giemsa-stained sections (**A7**) and as light yellow in van Gieson-stained sections (**B7**), respectively (green arrows). This finding is strong direct proof of the osteo-inductive feature of the hydrogel component, inducted through the generation of foreign body giant cell fibrous granulomatous inflammation. This inflammation is demonstrated in image (**B6**), where the βTCP-H bone substituent can be seen (blue arrow), alongside inflammatory mononuclear cells and a multinucleated giant macrophage (black arrow). Except for (**A7**) (Giemsa) and (**B7**) (van Gieson), all images in this figure depict HE-stained paraffin-embedded decalcified sections.

**At the 1st month of observation after implant treatment with H and βTCP-H materials,** early lateral and intra-lesional re-ossification induction was observed, accompanied by the presence of hydrogels (H) and βTCP-H (Figure 6A1,A2,B1,B2). Large pools of hydrogels (H) and βTCP-H (Figure 6A1,B1) were observed, surrounded by inflammatory fibrous tissues, coupled with intense ossification processes (Figure 6A2,B2, left). Budding new bone was covered by several layers of osteoblasts (Figure 6A2,B2, arrows), which resulted in osteoid matrix formation. This matrix originated from the remaining bone and independently from the lateral bone, inside the lesion, as evidenced by the small newly formed bony islets in Figure 6B1. Additionally, due to the methacrylated PGA contents in both the hydrogel (H) and βTCP-H exogenous bone substituent materials, they both exhibited characteristic birefringence under polarizing microscopy when present in the tissues (Figure 6A-B^i^ insert). Although these materials have been shown to be non-toxic [15], they appeared to induce chronic fibrous granulomatous inflammation. This was highlighted by the presence of epithelioid histiocytes and multinucleated giant macrophages (also called foreign body giant cells), which in turn underwent calcification (Figure 6B1, dark blue arrow and Figure 6B2) and ossification.

**At the 3rd month of postoperative observation after implant treatment with H and βTCP-H materials**, ongoing lateral and intra-lesional ossification was observed, accompanied by persistent H and βTCP-H within the fibrous granulomatous inflammatory tissues in both groups, with many multinucleated macrophages present (Figure 6A3,A4,B3,B4). By this time, the remaining non-eliminated hydrogel and βTCP-containing hydrogel (Figure 6A3 left lower corner; Figure 6B3 right) showed significantly decreased birefringence, indicating partial tissue processing and metabolism. Nevertheless, granulomatous inflammation still persisted in association with the remaining exogenous materials, highlighted by the presence of “foreign body” multinucleated giant macrophages (Figure 6A3, and Figure 6A3^i^-B3^i^ insert). Furthermore, reparative lateral and multifocal intralesional ossifications were prominent in both groups, accompanied by osteoblast proliferation (Figure 6B3,A4). However, the βTCP-containing hydrogel-treated bone defects appeared to contain more mature newly formed intralesional bone islets (Figure 6B4), which were preceded by osteoid matrix formation (Figure 6B4). Since osteoid matrix may show morphologic features similar to amyloid, we also used Congo Red staining, which was negative for amyloid deposition.

**At the 6th month of observation after implant treatment with H and βTCP-H materials**, advanced lateral and intra-lesional ossification was observed, resulting in substantial unifying bony reconstruction of the defects. However, fibrous tissues and areas inside of the newly formed bones harbored some amounts of non-eliminated residual H and βTCP-H materials, which were clearly visible under the microscope (Figure 6A5–A7,B5–B7). Although large coalescing new bone islets had developed by this time and became connected with the lateral and intralesional ones, substantial amounts of H and βTCP-H remained in the tissues (Figure 6A5,B6), contributing to the persistent chronic active granulomatous inflammation (Figure 6A6,B6 blue arrow). However, at this time, most of the lateral and intralesional new bones had undergone terminal maturation, as indicated by the organized lamellar hypocellular extracellular matrices and bone marrow formations, respectively (Figure 6A7,B7). Remarkably, some of the H and βTCP-H remnants were found intraosseously within the newly formed bones, These remnants were identified as blue areas on Giemsa-stained sections (Figure 6A7) and as light-yellow areas on van Gieson-stained sections (Figure 6B7), respectively (green arrows). This finding provides strong direct evidence of the hydrogel’s powerful osteo-inductive properties, most likely through the generation of foreign body giant cell fibrous granulomatous inflammation. Such inflammation is depicted in image Figure 6. In Figure 6B6, residues of the unmetabolized βTCP-H bone substituent particles can be seen (blue arrow), accompanied by chronic inflammatory mononuclear cells and a giant multinucleated macrophage (black arrow).

In conclusion, the presence of intraosseous hydrogel-containing graft materials, i.e., located inside the newly formed bone tissues (Figure 6A7,B7, arrows), unambiguously and irrefutably provides evidence for the osteo-inductive engineering features of both H alone and βTCP-H during the process of bone defect reconstruction.

#### 2.1.3. Morphometric Assessment of Time-Dependent Re-Ossification in Rat Calvaria Bone Defect

As shown in Table 1, even the untreated (control) calvaria bone defects exhibited time-dependent gradual solidification, predominantly with fibrous scar tissue formation and lateral ossification. The latter resulted in 57% compact new bone by the 6th month of observation. However, when using aerogel (AE) or hydrogel (H) to fill the bone defect, more powerful osteo-induction could be detected, arising not only from the lateral bone edges of the defect but also within the lesion. This resulted in 72.8% and 76.9% new bone production within the defect, respectively, by the end of the observation period. The rest of the defects mainly contained collagen-rich fibrous (scar) tissue. Remarkably, when using beta-tricalcium phosphate (βTCP)-containing aerogel (βTCP-AE) or hydrogel (βTCP-H), substantially more dynamic and robust intralesional re-ossification occurred, resulting in over 80% new bone formation embedded in fibrous tissue within the defect by the 6th month postoperatively.

### 2.2. Discussion

Exogenous grafting (alloplastic) materials are often used in reconstructive bone surgery to fill and replace bone defects or to induce facilitated re-ossification for speedy tissue remodeling. The aim is to achieve complete repair and restore physiological functions [16,17,18,19]. Titanium-based metals are among the most preferred materials in orthopedic and reconstructive oral surgeries [20] and dental implants [21]. However, alloplastic ceramics and hydrogels with various chemical additives are believed to exhibit more osteo-inductive features. These materials can be applied in large bone defects, providing temporal scaffolding within the bone injury while stimulating the process of ossification not only at the lateral edges, i.e., the interface of the remaining internal bone surfaces, but also inside the lesion, independent of the peripheral presence of the regenerating osseous tissues.

In this respect, the present paper provides a comprehensive review of histopathological follow-up of osseous tissue defect repairs mediated by mesoporous silica-based aerogel (AE) type ceramics and methacrylated PGA-based hydrogel (H), including their beta-tricalcium phosphate (βTCP)-containing counterparts (βTCP-AE and βTCP-H). The duration of the analysis was 6 months, which is the widely accepted time frame for complete wound healing and resolution. In previous research, we demonstrated that these compounds are non-toxic, well-tolerated, and applicable as biocompatible osteo-inductive templates to facilitate bone tissue reconstruction [13,15,22,23,24]. The silica and the MPGA contents of these alloplastic materials allowed us to follow their tissue presence and, in turn, assess their rate of metabolism at grafting sites by means of a polarizing microscope, as both exhibit birefringence under polarized lights. The following summarized observations were obtained:

In bone defects within a rat calvaria model that were left untreated (control), re-ossification exclusively started at lateral regions from the remaining bone’s edges. The process of regenerative osteogenesis occurred through osteoblast activation in a budding pattern, facilitated by mesenchymal cell debris from the injured tissue and components of extravasated blood, which induced granulation tissue (GT) formation via angiogenesis with fibroblasts and mixed inflammatory cells. The cellular density of GT was highest during the 1st month. Afterward, a gradual decay in the leukocyte and capillary densities was observed, while fibrosis increased by the 3rd month and continued thereafter. This led to the solidification and filling of the defect, predominantly with connective tissues. Apart from some calcifications, up to the third month of observation, no unambiguous osseous tissue formation was found inside the untreated lesions that could be considered independent from the lateral bone formations. By the 6th month, only moderate amounts of small bone islets were formed with osteoid matrix, (closely mimicking dystrophic ossification) which appeared independent of the lateral ossification. Nevertheless, the lateral ossification resulted in 57% osseous encroachment of the defect, without treatment.

In contrast, all of the mesoporous ceramics and polymers, including the native aerogel (AE), hydrogel (H), and their β-tricalcium phosphate (βTCP)-containing counterparts (βTCP-AE and βTCP-H), that were inserted into the bone defects characteristically induced chronic active granulomatous inflammation. This inflammation was accompanied by epithelioid histiocyte clusters and multinucleated giant macrophages, persistent in decreasing intensity throughout the entire observational period. This was preceded by nonspecific (injury-associated) transient inflammation and GT formation in the 1st month. In addition to the lateral ossifications that were detected with a control-comparable pattern in all sample types, multiple and intense osseous foci developed inside the lesions as early as the 1st month. These foci grew progressively in a multi-directional manner, reflecting the osteo-inductive effects of all compounds. Both βTCP-containing bone substituents generated larger amounts and more mature new bones inside the defects. Nevertheless, only 72.8% to 76.9% of the bone defect treated with AE and H and 80.5% to 82.9% of those treated with the βTCP-containing counterparts were re-ossified by the 6th and final month of observation. Remarkably, by this time, significant amounts of hydrogel remained uneliminated from the tissues, and trace amounts of silica of AE remnants were still detectable by means of polarizing microscopy, indicating these as causative agents of the persistent fibrous granulomatous inflammation. The overall main conclusion is that when using silica or MPGA-based hydrogel bone substituents to restore a bone deficit, robust osteo-induction occurs through self-limited chronic ossifying fibrous granulomatous inflammation (i.e., granuloma formation), resulting in osseointegration. Nevertheless, 100% re-ossification most likely takes more than 6 months,; however, by this time, this degree of osteo-fibrous solidification with tight osseointegration appears to provide functionally well-suited bone repair.

**Figure 7:**
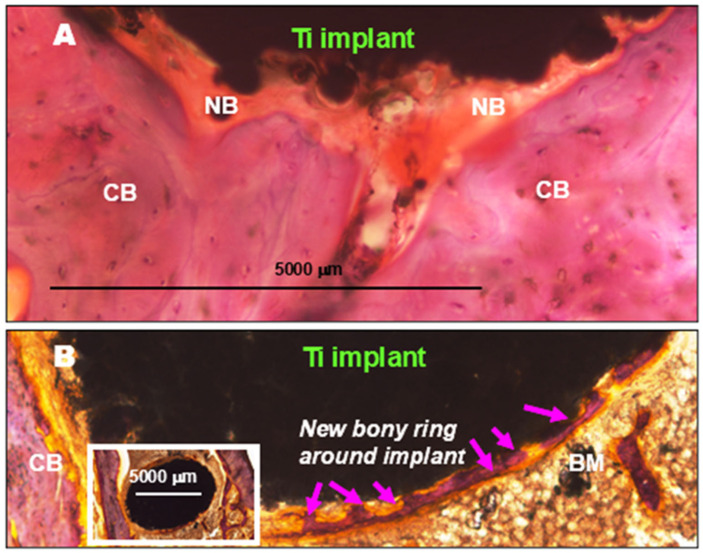
Ground specimens showing portions of cross-sectioned titanium implants surrounded by the recipient bone, 3 months after operation, and stained with hematoxylin–eosin. (**A**) A dog’s pre-molar compact bone (CB) with a gyroid titanium implant tightly integrated into the newly formed bone tissue (NB). (**B**) Cross section of a sheep’s bone femur condyle (insert), and a portion of this image at higher magnification showing the formation of a new osseo-fibrous bony ring within the bone marrow (BM). This integrated tightly with the implant’s surface (arrows) by the 3rd month. (Adapted from our archived tissue files obtained from unpublished earlier data of Ref. [25]).

Contrary to this morphologic pattern, titanium implants without a surface coating do not lead to granulomatous inflammation during the process of osseointegration. On the other hand, based on our archived tissue sections, a complete callus-like ossifying ring around implants readily developed in a much shorter time, i.e., by the 3rd month, in dogs’ alveolar bones and in sheep’s femur condyle (Figure 7) [25]. The force-activated ossification induced by solid metal implants and the injury-associated reactive inflammatory edema fluid containing mesenchymal cellular remnants (containing surviving stem cells) lead to powerful contact osteo-induction. This promotes tight connection of the bone with the implants for optimal integration, as confirmed by others [26,27,28].

Our histopathology findings on the in vivo effectiveness of silica aerogels and methacrylate-based hydrogels in bone regeneration cannot be directly compared with the osteogenic capacities of other bone-substituting bioactive materials reported so far [29,30,31,32]. This is because of the different chemical compositions and the variable experimental or clinical systems, as well as the typically shorter follow-up time for complete ossification. Additionally, it should be noted that ranking bone substituent scaffolds based on their re-ossification capacities is difficult, given the complex medical requirements that exist in accordance with disease types and specific needs in patient management. Regarding the persistent granulomatous inflammation observed in this study, it is typically induced by the presence of silica and methacrylate components of the applied scaffolds in the tissue. Due to their birefringencies, these compounds can be easily identified and followed under a polarizing microscope; therefore, their clearance from the tissue can be assessed [33,34]. Although they are inert and nontoxic to living tissues, they are recognized as foreign bodies by the host. This induces a typical primary chronic (sterile) fibrous granulomatous (nodular) reaction with clusters of activated mononucleated cells and occasional multinucleated (giant) macrophages, which engulf, eliminate, and localize the “invader”, also known as the foreign body giant cell reaction. Essentially, this type of inflammation represents the so-called type IV hypersensitivity (i.e., immune-mediated cellular) tissue reaction, in which activated macrophages/histiocytes are the key elements. These cells further activate other cells, including fibroblasts and, in osseous environments, osteoblasts, to induce osteogenesis until the bony repair is completed and the crystals are eliminated. The cytokines and other cell-activating or signaling factors involved in this process require further investigation.

## 3. Conclusions

According to the evidence-based comparative microscopic tissue analysis of rat calvaria bone defects grafted with bioactive mesoporous silica aerogel (AE) or hydrogel (H) with and without β-tricalcium-phosphate (βTCP) contents to facilitate ossifying repair, the following conclusions can be drawn:

(1) Compared to the untreated control cases, all of the above bone substituents demonstrated well-tolerated healing processes with significant stimulated osseous restoration. This was preceded by control-comparable injury-associated common transient non-specific inflammation, followed by capillary-rich granulation tissue formation.
(2) Without treatment, i.e., no bone defect substitution (control), the re-ossification exclusively started along the lateral edges of the bony defect. This regenerative osteogenesis, driven by osteoblasts, could be detected as early as the 1st month after the operation. While lateral ossification continued throughout the observational time, although with gradually decreasing intensity along with increasing scarring, by the 6th month, only moderate amounts of intralesional new bone islets had formed within the fibrous tissues, independently of the lateral ossification.
(3) In contrast, all of the applied bioactive bone substituents, including AE, H, and their βTCP-containing counterparts, used to fill the bone defects not only induced robust lateral re-ossification (arising from the remaining circular bone edges of the defect), but early and rapid intralesional multifocal ossification as well. This resulted in well-suited substantial osseous bone remodeling for restoration by the end of the observation period. The facilitated osteo-induction is directly related to the presence of AE and H crystal particles in the tissue as foreign body components. These particles characteristically induce low-grade chronic protracted granulomatous inflammation to recruit T cells, alternatively activated macrophages, and multinucleated histiocytes (“foreign body giant cells”) for elimination of the “invaders”. According to our findings, this process did not appear to be complete by the 6th month of follow-up. Nevertheless, it is noteworthy that such inflammation may have some beneficial effects in certain conditions. In a bony environment, activated inflammatory cells activate fibroblasts and likely stems cells capable of osteogenic differentiation within the injured bony lesion, facilitating ossification and subsequently, bone reconstruction.
(4) Among the applied scaffolds, the βTCP-containing AE and H composite materials proved to be the most powerful in terms of their osteo-inductive capacities for bone repair. However, the methacrylate component of the hydrogel, irrespective of βTCP content, showed the slowest rate of tissue clearance from the healing bone defect, potentially sustaining the persistent low-grade chronic inflammation.

## 4. Materials and Methods

### 4.1. Preparation of Scaffolds

#### 4.1.1. Preparation of Mesoporous Silica Aerogel and βTCP-Aerogel

**Figure 8:**
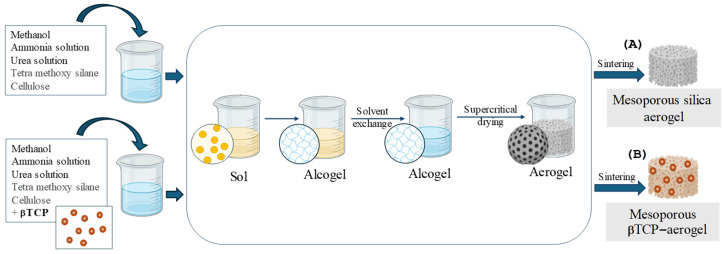
Schematic illustration of the preparation of mesoporous silica aerogel (**A**) and βTCP-aerogel (**B**).

Silica aerogel (AE) and its βTCP-containing counterpart (βTCP- AE) were made using the base-catalyzed sol-gel technique [35], according to the upgraded methods established in our laboratories and published in detail earlier [15,22,23,36]. Briefly, the silica aerogel fabrication involved the following distinct steps: (1) the process of gelation (sol-gel transition), (2) network development by aging, and (3) gel-to-aerogel transformation using supercritical carbon dioxide drying of the wet gel. While the preparation of pristine silica aerogel is rather straightforward, its heterogeneous composite with βTCP requires careful control of viscosity, mixing, and stirring to prevent sedimentation of the higher-density calcium phosphate component. Careful selection of the reaction solvents, the addition of hydrogen-bonding additives, including the urea, and accurate titration of the base catalyst are the key steps for successful implementation of this method, as described in the literature [37]. Before any biomedical applications, the super-critically dried products required an elevated-temperature furnace treatment and sintering to reach their optimal purity and mechanical strength [15,37]. The physicochemical characteristics of the fabricated biomaterials have been described in our previous papers [15,22,23,36]. Subsequently, pre-powdered and sterilized AE and βTCP-AE samples were used to fill cavities with a diameter of 8 mm and a depth of approximately 1.5 mm, according to the standard surgical technique. The aerogels were initially sterilized with in situ generated ozone (O_3_) for 10 min using an OzoneDTA O_3_ generator (Apoza Enterprise, Taiwan, ROC), followed by 50 min of UV light exposure inside a microbiological safety cabinet (Bioair, Topsafe 1.2, EuroClone, Siziano, Italy). The preparation of the mesoporous silica aerogel and βTCP-aerogel scaffolds is schematically illustrated in Figure 8.

#### 4.1.2. Preparation of Methacrylated Polyglutamic Acid-Based Hydrogel and βTCP-Hydrogel

**Figure 9:**
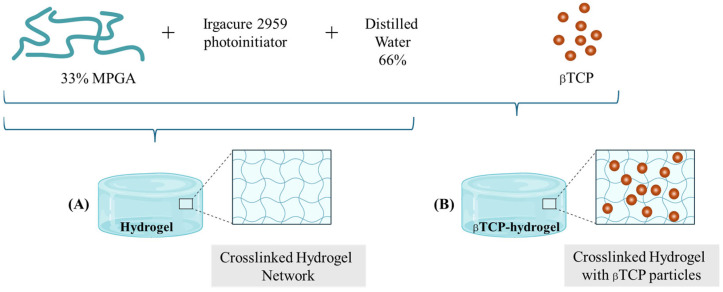
Schematic illustration of the preparation of methacrylated polyglutamic acid-based hydrogel (**A**) and βTCP-hydrogel (**B**).

Poly-γ-glutamic acid (PGA)-based hydrogels were prepared from methacrylated poly-γ-glutamic acid (MPGA) and Irgacure 2959 (~99%, Sigma-Aldrich, St. Louis, MO, USA) photoinitiator (8 n/n% calculated for the methacryloyl group) through a free radical polymerization reaction. The production of MPGA has been reported earlier. Briefly, PGA was activated by 1-[3-(dimethylamino) propyl]-3-ethyl carbodiimide hydrochloride (Carbosyth Limited, Compton, Berkshire, UK), and the methacrylation reactions were carried out using 2-aminoethyl methacrylate hydrochloride (90%) [13,24]. Then, hydrogels consisting of 33 *w*/*w*% MPGA and 66% distilled water were photopolymerized for 1 min using a Bluephase 20i (Ivoclar Vivadent AG, Schaan, Liechtenstein) dental polymerization unit (hand lamp, with 2000 mW/cm^2^). A custom-made cylindrical Teflon mold measuring 1.5 mm in depth and 8 mm in diameter was used to create the samples. The sterilization process of the samples was performed under a laminar flow box (Bioair, Topsafe 1.2, EuroClone, Italy) using UV-lighting methods for 50 min, first on one side and then on the other. The samples were stored in a humidity chamber until application. The β-tricalcium phosphate 4 μm ≥ 80 m^2^g (βTCP) (Sigma-Aldrich, St. Louis, MO, USA)-containing samples were produced using the same method, but the hydrogels were modified with 18.5 *w*/*w* % βTCP content. The components of the methacrylated polyglutamic acid- based hydrogel and the βTCP-hydrogel, along with the structural scheme of the gels, are shown in Figure 9.

### 4.2. Animals

For the “critical size model”, 3-month-old male Wistar rats weighing 250–300 g were used for the in vivo study on calvaria bones. Originally, the sample groups included *n* = 6 per observation time point, but due to technical reasons during the surgeries and tissue processing, a minimum of 4 animals/time point were ultimately included in each treatment group. The animals were kept in a conventional animal house at a controlled temperature (26 °C ± 2° C) and humidity (55 ± 5%). Artificial lighting was provided in automatically controlled 12 h circadian cycles. The experimental protocol was approved by the Institutional Review Board for the “Animal Care and Use Committee” of the University of Debrecen (7/15-2/DEMÁB). The animals were kept on a standard diet and allowed to drink water ad libitum (VRF1 rodent chow, Akronom Ltd., Budapest, Hungary). All of the procedures of the studies were conducted in accordance with Hungarian Law and the Helsinki Declaration.

### 4.3. Surgical Procedures

**Figure 10:**
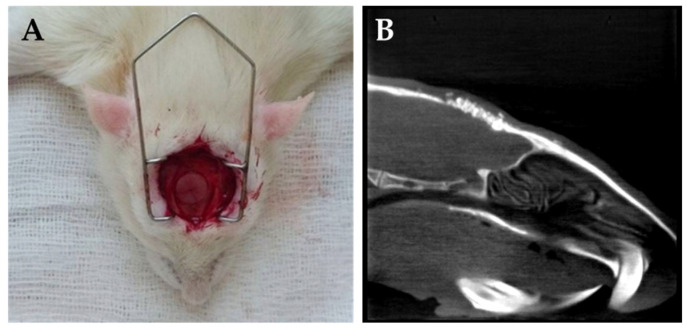
Surgery (**A**) for the implantation of βTCP-AE into a rat calvaria bone defect. (**B**) Imaging of βTCP-AE scaffolds using cone beam computed tomography.

All operations were carried out under sterile conditions. The rats were anesthetized through intraperitoneal injection of ketamine (CP-Ketamin 10%, Prod-ulab Pharma B.V., Raamsdonksveer, Holland), in combination with xylazine (Xylazin 2%, CP-Pharma, Burgdorf, Germany) at a 2:1 ratio (0.5–0.55 mL per animal) [15]. To evaluate bone regeneration, the “rat critical size calvaria defect” model was used according to the instructions for the step-by-step surgical procedures, published in *Nature Protocols* by Spicer P.P. et al. [29], and as described in our previous paper [15]. Briefly, after shaving the dorsal part of the cranium, a sagittal aseptic midline incision was made, running through the skin, the underlying muscle, and the periosteum, to reach the calvarium. This was followed by a gentle preparation of the periosteal membrane from the parietal bone. Using an 8 mm diameter sterile dental trephine bur, the bone was exposed in the midline of the parietal region and gradually cut and thinned at a low speed. The full thickness of the bone was then removed up to the dura, which was left intact to prevent any brain injury. The bone defect was then cleaned with intense saline irrigation. After inserting the various types of biomaterials (Figure 10) (or leaving the site empty for controls), the periosteum and the overlying skin were then closed in two layers with resorbable sutures. To prevent postoperative infections, the animals received antibiotics for 5 days, as previously described [15]. After an uneventful postoperative period, at the indicated times (1st month, 3rd month, and 6th month), the animals were sacrificed with an ip injection of an overdose of anesthetics. Then, the calvaria bone with the defect was removed for histopathological analyses.

### 4.4. Tissue Processing for Microscopic Analyses

After transcardial saline perfusion to reduce the presence of blood in the specimen, the skull containing each bone defect was removed en bloc, together with the periosteum, some overlying muscle layer, and a rim of normal surrounding bone (specimen number: 4/group/time point). Following fixation in 10% neutral buffered formaldehyde (pH 7.4) for 3 days at room temperature, the tissue samples were rinsed in saline. In addition to the hydrogel-treated groups, one specimen from each group was saved for resin embedding to prepare ground specimen sections (see below). The remaining three bone specimens (per group per time point) were subjected to de-calcination using 14% EDTA (ethylene diamine tetraacetate) containing 3% formalin at room temperature until the tissue became rubbery and easily cuttable. After orientation, the specimens were then sagittally halved to produce 2-2 samples, providing side views of the healing bone defects. Following dehydration in graded ethanol and acetone, all decalcified samples were paraffin-embedded. After serial step-sectioning and mounting onto “X-tra^TM^” Adhesive glass slides (Leica Biosystem, Petersborough, UK), the tissues were stained with hematoxylin–eosin (HE), picrosirius-based van Gieson (vG), and Masson trichrom (MTri) for collagen detection, as well as Giemsa (G), von Kossa (vK), and Congo Red (C-R), respectively, with standard methods that we have previously described [33,38,39]. In addition to scanning and digitalizing the stained tissue sections (see below), they were analyzed under a Leica DM2500 microscope equipped with a polarizing device and a Leica DFC500 camera (Leica Microsystems CMS35578, Wetzlar, Germany) for photography.

### 4.5. Ground Section Preparation of Non-Decalcified Bone Defects

The untreated (control) and the different types of bone substituents containing formalin-fixed tissue blocks from the rat calvaria bone defect experiments (see above) were used to process thin-ground sections. After rinsing in saline, the samples were dehydrated in increasing concentrations of ethanol and xylene. Native bone specimens were embedded in epoxy resin using the EpoFix kit (Strues, Ballerup, Denmark). Then, the blocks were sliced into 2 mm thick crude sections using a hard tissue microtome (Leitz 1600, Nussloch, Germany) and thermo-plastically adhered (Crystalbond 509 mounting adhesive, SPI Supplies, West Chester, PA, USA) onto poly-L-lysine coated glass slides (Sigma). Finally, they were polished to 10–15 µm sections. Following treatment with 95% ethanol saturated with sodium hydroxide, the polished slides were stained with Gill’s hematoxylin and eosin Y-phloxine B, as previously described [40].

### 4.6. Immunohistochemistry (IHC)

Representative decalcified and de-paraffinized sections from the treated and control paraffin-embedded samples were used for peroxidase-based immunohistochemical labeling, as described in our earlier papers [33,38,39,41]. Briefly, after antigen retrieval at pH6.0 or pH9.0 (as suggested by the vendors for each marker), the following primary antibodies were used: rabbit monoclonal antibody (R-mab) to Ki-67 (for the assessment of cellular activities; Abcam, UK); R-mab to carboxypeptidase-M (CPM; Abcam, UK) for granuloma histiocytes [19]. After incubating the sections with the appropriately diluted primary antibodies at room temperature for 1 h (as suggested by the vendor), an EnVision^+^-HRP detection kit (Dako, Glostrup, Denmark) was used with VIP (purple) or DAB (brown) peroxidase substrates. Both the conventionally stained and the immune-labelled sections were then digitalized using the Panoramic MIDI digital slide scanner (3D-Histotech-Zeiss, Budapest, Hungary) equipped with a Hitachi 3CCD progressive scan color camera (HV-F22CL). Comparative image analyses were then performed using the HistoQuant application of Panoramic viewer software 1.15.2 (3D-Histotech), as previously described [41].

### 4.7. Morphometric Assessment of Re-Ossification

The percentage of ossification in the bone defect was estimated based on the amounts of new bone and osteoid matrix formation over the observational period using the scanned and digitalized van Gieson-stained slides, with measurements collected from the superficial, middle, and deep levels, respectively, of three samples/group. The average (%) values were then calculated for each time point as the mean percentages of the corresponding available defect size found under the microscope.

## Figures and Tables

**Table 1:** Mean percentages and standard deviations of ossifications in rat calvaria bone defects filled with bone substituents over time.

	Control (Untreated) (±SD)	Aerogel (±SD)	βTCP-Aerogel (±SD)	Hydrogel (±SD)	βTCP-Hydrogel (±SD)
1 Month	11.8% ± 3.9	15.0% ± 5.7	19.4% ± 6.3	26.7% ± 7.1	30.0% ± 5.0
3 Months	23.1% ± 8.8	40.0% ± 13.5	53.9% ± 14.1	60.5% ± 13.8	65.0% ± 9.0
6 Months	57.0% ± 9.5	72.8% ± 10.6	80.5% ± 11.6	76.9% ± 12.5	82.8% ± 10.1

## Data Availability

The raw data supporting the conclusions of this article will be made available by the authors, without undue reservation.

## Abbreviations

AE	aerogel
βTCP	β-tricalcium-phosphate
βTCP-AE	β-tricalcium-phosphate-containing aerogel
βTCP-H	β-tricalcium-phosphate-containing hydrogel
BM	bone marrow
CB	compact bone
C-R	Congo Red
G	Giemsa
H	hydrogel
HE	hematoxylin–eosin
MPGA	methacrylated poly-γ-glutamic acid
MTri	Masson trichrom
NB	new bone
vG	picrosirius-based van Gieson
vK	von Kossa

## References

[1] Jin W., Chu P.K. (2019). Osseointegration. Encyclopedia of Biomedical Engineering.

[2] Hudecki A., Kiryczyński G., Łos M.J., Łos M.J., Hudecki A., Wiecheć E. (2019). Biomaterials, Definition, Overview (Chapter 7). Stem Cells and Biomaterials for Regenerative Medicine.

[3] Khan W.S., Rayan F., Dhinsa B.S., Marsh D. (2012). An osteoconductive, osteoinductive, and osteogenic tissue-engineered product for trauma and orthopedic surgery: How far are we?. Stem Cells Int..

[4] Pérez-Moreno A., Piñero M., Fernández-Montesinos R., Pinaglia-Tobaruela G., Reyes-Peces M.V., Mesa-Díaz M.d.M., Vilches-Pérez J.I., Esquivias L., de la Rosa-Fox N., Salido M. (2023). Chitosan-Silica Hybrid Biomaterials for Bone Tissue Engineering: A Comparative Study of Xerogels and Aerogels. Gels.

[5] Lázár I., Čelko L., Menelaou M. (2023). Aerogel-Based Materials in Bone and Cartilage Tissue Engineering—A Review with Future Implications. Gels.

[6] Boda R., Lázár I., Keczánné-Üveges A., Bakó J., Tóth F., Trencsényi G., Kálmán-Szabó I., Béresová M., Sajtos Z., Tóth E.D. (2023). β-Tricalcium Phosphate-Modified Aerogel Containing PVA/Chitosan Hybrid Nanospun Scaffolds for Bone Regeneration. Int. J. Mol. Sci..

[7] Hegedűs C., Czibulya Z., Tóth F., Dezső B., Hegedűs V., Boda R., Horváth D., Csík A., Fábián I., Tóth-Győri E. (2022). The Effect of Heat Treatment of β-Tricalcium Phosphate-Containing Silica-Based Bioactive Aerogels on the Cellular Metabolism and Proliferation of MG63 Cells. Biomedicines.

[8] Huang G.J., Yu H.P., Wang X.L., Ning B.B., Gao J., Shi Y.Q., Zhu Y.J., Duan J.L. (2021). Highly porous and elastic aerogel based on ultralong hydroxyapatite nanowires for high-performance bone regeneration and neovascularization. J. Mater. Chem. B.

[9] Zhao Y., Cheng C., Wang X., Yuan Z., Sun B., El-Newehy M., Abdulhameed M.M., Fang B., Mo X. (2024). Aspirin-Loaded Anti-Inflammatory ZnO-SiO_2_ Aerogel Scaffolds for Bone Regeneration. ACS Appl. Mater. Interfaces.

[10] Weng L., Boda S.K., Wang H., Teusink M.J., Shuler F.D., Xie J. (2018). Novel 3D Hybrid Nanofiber Aerogels Coupled with BMP-2 Peptides for Cranial Bone Regeneration. Adv. Healthc. Mater..

[11] Maisani M., Pezzoli D., Chassande O., Mantovani D. (2017). Cellularizing hydrogel-based scaffolds to repair bone tissue: How to create a physiologically relevant micro-environment?. J. Tissue. Eng..

[12] Chen C., Li Z., Xu C., Kang M., Lee C.S., Aghaloo T., Lee M. (2024). Self-Assembled Nanocomposite Hydrogels as Carriers for Demineralized Bone Matrix Particles and Enhanced Bone Repair. Adv. Healthc. Mater..

[13] Bakó J., Vecsernyés M., Ujhelyi Z., Kovácsné I.B., Borbíró I., Bíró T., Borbély J., Hegedűs C. (2013). Composition and characterization of in situ usable light cured dental drug delivery hydrogel system. J. Mater. Sci. Mater. Med..

[14] Győri E., Fábián I., Lázár I. (2017). Effect of the Chemical Composition of Simulated Body Fluids on Aerogel-Based Bioactive Composites. J. Compos. Sci..

[15] Hegedüs V., Kerényi F., Boda R., Horváth D., Lázár I., Tóth-Győri E., Dezső B., Hegedüs C. (2018). β-Tricalcium phosphate silica aerogel as an alternative bioactive ceramic for the potential use in dentistry. Adv. Appl. Ceram..

[16] García-Gareta E., Coathup M.J., Blunn G.W. (2015). Osteoinduction of bone grafting materials for bone repair and regeneration. Bone.

[17] Habibovic P., de Groot K. (2007). Osteoinductive biomaterials--Properties and relevance in bone repair. J. Tissue Eng. Regen. Med..

[18] Zhao Y., Duan W., Zhu B., Chen Y., Zhu Y., Martin-Saldaña S., Xiao Z., Liu X., Feng L., Ren Y. (2025). Nanozyme-Engineered Hyaluronic Acid Adhesives Loading Platelet-Rich Plasma for Multilayered Osteoarthritis Treatment with Pain-Relief Effect. Adv. Funct. Mater..

[19] Luo L., Gong Y., Yan L., Bu Y. (2024). Sponge as Scaffolds in Bone and Cartilage Tissue Engineering. Chem. Res. Chin. Univ..

[20] Gareb B., Van Bakelen N.B., Vissink A., Bos R.R.M., Van Minnen B. (2022). Titanium or Biodegradable Osteosynthesis in Maxillofacial Surgery? In Vitro and In Vivo Performances. Polymers.

[21] Pandey C., Rokaya D., Bhattarai B.P. (2022). Contemporary concepts in osteointegration of dental Implants. BioMed Res. Int..

[22] Lázár I., Bereczki F.H., Manó S., Daróczi L., Deák G., Fábián I., Csernátony Z. (2015). Synthesis and study of new functionalized silica aerogel poly(methyl methacrylate) composites for biomedical use. Polym. Compos..

[23] Lázár I., Fábián I. (2017). A continuous extraction and pumpless supercritical CO_2_ drying system for laboratory-scale aerogel production. Gels.

[24] Lazar I., Fabian I. (2011). Process for the Preparation of Composite Silica Alcogels, Aerogels and Xerogels, Apparatus for the Continuous Implementation of the Process, and New Composite Silica Alcogels, Aerogels and Xerogels. WO2013061104.

[25] Kovács Á.É., Csernátony Z., Csámer L., Méhes G., Szabó D., Veres M., Braun M., Harangi B., Serbán N., Zhang L. (2023). Comparative Analysis of Bone Ingrowth in 3D-Printed Titanium Lattice Structures with Different Patterns. Materials.

[26] Franchi M., Orsini E., Trire A., Quaranta M., Martini D., Piccari G.G., Ruggeri A., Ottani V. (2004). Osteogenesis and morphology of the peri-implant bone facing dental implants. Sci. World J..

[27] Guglielmotti M.B., Olmedo D.G., Cabrini R.L. (2000). Research on implants and osseointegration. Periodontology.

[28] Terheyden H., Lang N.P., Bierbaum S., Stadlinger B. (2012). Osseointegration--Communication of cells. Clin. Oral Implant. Res..

[29] Spicer P.P., Kretlow J.D., Young S., Jansen J.A., Kasper K.F., Mikos A.G. (2012). Evaluation of bone regeneration using the rat critical size calvarial defect. Nat. Protoc..

[30] Dec P., Modrzejewski A., Pawlik A. (2023). Existing and Novel Biomaterials for Bone Tissue Engineering. Int. J. Mol. Sci..

[31] Yi H., Ur Rehman F., Zhao C., Liu B., He N. (2016). Recent advances in nano scaffolds for bone repair. Bone Res..

[32] Todd E.A., Mirsky N.A., Silva B.L.G., Shinde A.R., Arakelians A.R.L., Nayak V.V., Marcantonio R.A.C., Gupta N., Witek L., Coelho P.G. (2024). Functional Scaffolds for Bone Tissue Regeneration: A Comprehensive Review of Materials, Methods, and Future Directions. J. Funct. Biomater..

[33] Tsakiris I., Torocsik D., Gyongyosi A., Dozsa A., Szatmari I., Szantó A., Soos G., Nemes Z., Igali L., Marton I. (2012). Carboxypeptidase-M is regulated by lipids and CSFs in macrophages and dendritic cells and expressed selectively in tissue granulomas and foam cells. Lab. Invest..

[34] Sellamuthu R., Umbright C., Roberts J.R., Young S.H., Richardson D., McKinney W., Chen B.T., Li S., Kashon M., Joseph P. (2017). Molecular mechanisms of pulmonary response progression in crystalline silica exposed rats. Inhal. Toxicol..

[35] Kistler S. (1931). S Coherent expanded aerogel and jellies. Nature.

[36] Lázár I., Szabó H.J. (2018). Prevention of the Aggregation of Nanoparticles during the Synthesis of Nanogold-Containing silica aerogels. Gels.

[37] Kuttor A., Szalóki M., Rente T., Kerényi F., Bako J., Fabian I., Lazar I., Jenei A., Hegedus C. (2014). Preparation and application of highly porous aerogel-based bioactive materials in dentistry. Front. Mater. Sci..

[38] Jókay I., Soós G., Répássy G., Dezső B. (1998). Apoptosis in the human inner ear. Detection by in situ end-labeling of fragmented DNA and correlation with other markers. Hear. Res..

[39] Szántó A., Bálint B.L., Nagy Z., Barta E., Dezső B., Pap A., Széles L., Póliska S., Oros M., Evans R.M. (2010). STAT6 Transcription Factor is a Facilitator of the Nuclear Receptor PPARγ-Regulated Gene Expression in Macrophages and Dendritic Cells. Immunity.

[40] Murice-Lambert E., Banford A.B., Folger R.L. (1989). Histological preparation of implanted biomaterials for light microscopic evaluation of the implant-tissue interaction. Stain Technol..

[41] Szabo K., Papp G., Dezso B., Zeher M. (2014). The histopathology of labial salivary glands in primary Sjogren’s syndrome: Focusing on follicular helper T cells in the inflammatory infiltrates. Mediat. Inflamm..

